# Synthetic extracellular volume fraction as an imaging biomarker of the myocardial interstitium without blood sampling: a systematic review and meta-analysis

**DOI:** 10.1016/j.jocmr.2025.101889

**Published:** 2025-03-24

**Authors:** Naofumi Yasuda, Shingo Kato, Nobuyuki Horita, Ryusuke Sekii, Shungo Sawamura, Hiroaki Nagase, Daisuke Utsunomiya

**Affiliations:** a Department of Diagnostic Radiology, Yokohama City University Graduate School of Medicine, Yokohama, Kanagawa, Japan; b Chemotherapy Center, Yokohama City University Hospital, Yokohama, Kanagawa, Japan; c Department of Cardiology, Yokohama Hodogaya Central Hospital, Yokohama, Kanagawa, Japan

**Keywords:** Synthetic extracellular volume fraction, Synthetic hematocrit, Magnetic resonance imaging

## Abstract

**Background:**

The calculation of conventional extracellular volume fraction (ECV) requires blood hematocrit (Hct) measurement. Based on the relationship between Hct and blood T1 relaxivity for cardiac magnetic resonance (CMR), a synthetic ECV could be estimated without a blood sampling. The aim of this study was to evaluate the correlation and agreement in the quantification of synthetic ECV and laboratory ECV from conventional Hct measurements.

**Methods:**

Electronic database searches of PubMed, Web of Science Core Collection, Cochrane advanced search, and EMBASE were performed. The authors employed a meta-analysis using the generic inverse variance method with a random-effects model to estimate the summary correlation coefficient and mean absolute difference between synthetic and laboratory ECV.

**Results:**

Of 38 papers, 10 studies comprising 4492 patients were identified. Overall, there was an excellent correlation between synthetic ECV and laboratory ECV (0.95 [95% confidence interval (CI): 0.92 to 0.97]) at 1.5T CMR and (0.91 [95% CI: 0.86 to 0.94]) at 3.0T CMR. The pooled mean difference between synthetic ECV and laboratory ECV was 0.61% (95% CI: 0.23 to 0.98%, I^2^ = 0%, p for heterogeneity = 0.67) at 1.5T CMR and 0.24% (95% CI: −0.13 to 0.61%, I^2^ = 19%, p for heterogeneity = 0.25) at 3.0T CMR.

**Conclusion:**

This study is the first comprehensive systematic review and meta-analysis of synthetic ECV evaluation at CMR. Synthetic ECV demonstrated an excellent correlation with laboratory ECV, with a mean difference of less than 1%, and offers noninvasive and instantaneous quantification of the myocardial extracellular space without the need for blood sampling.

## Graphical abstract

**Figure:**
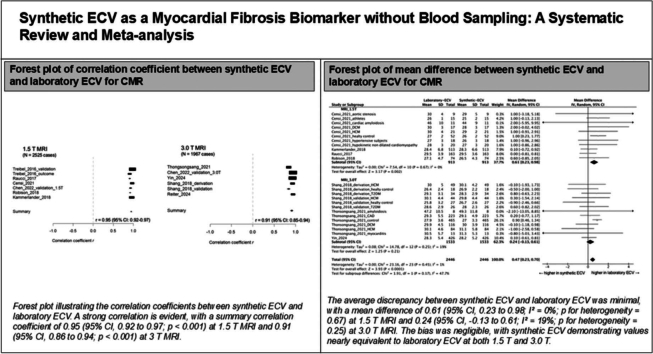

## 1. Introduction

Myocardial fibrosis is one of the characteristic pathological changes observed in many forms of cardiovascular diseases, a consequence of an increase in collagen formation in the extracellular matrix and myocyte cell death, and accurate assessment of this change is critical for diagnosis [1], [2]. Extracellular volume fraction (ECV) is a quantitative analysis derived from cardiovascular magnetic resonance (CMR) T1 mapping and well-established imaging biomarker for histological myocardial fibrosis, which differentiates normal from diseased myocardium, and associates with mortality and other adverse cardiocerebrovascular events [3], [4], [5], [6], [7].

However, calculation of ECV requires an accurate and timely assessment of blood hematocrit (Hct). The Society for Cardiovascular Magnetic Resonance Consensus Statement recommends that the Hct for ECV calculation should be obtained immediately before the CMR scan, if possible, otherwise within 24 h of the scanning [8]. Conventional laboratory determination of Hct via venipuncture presents several challenges, including increased cost, patient discomfort due to the invasive nature of the procedure, and practical inconveniences within the clinical settings. Additionally, it may cause difficulties for retrospective studies using ECV values because blood Hct is not routinely measured on the day of CMR scan in the setting of outpatients.

More recently, synthetic ECV has emerged as an alternative method for ECV quantification, removing the need for blood Hct measurement by utilizing the linear relationship between longitudinal relaxation rate (R1 = 1/T1) of blood and laboratory-measured Hct [9]. This eliminates the time and cost of obtaining a venous Hct sampling. Prior studies have shown good agreement between synthetic ECV values and laboratory ECV values at both 1.5T and 3.0T CMR in clinical cohorts [9], [10]. However, these studies are limited by their small sample size and it remained unclear whether the results could be replicated across different scanning protocols, scanners, and magnetic field strengths. Hence, we sought to perform a systematic review and meta-analysis integrating related articles to investigate the utility of synthetic ECV quantification with laboratory ECV as the reference standard.

## 2. Materials and methods

### 2.1 Search strategy and selection criteria

We used the methods suggested by the Cochrane Collaboration and met the reporting standards of the preferred reporting items for systematic reviews and meta-analyses (PRISMA) guideline of 2020 [11]. We conducted a database search using PubMed, Web of Science Core Collection, and EMBASE electronic database on June 7, 2024. synthetic extracellular volume fraction, synthetic ECV, cardiac magnetic resonance imaging (MRI), cardiac magnetic resonance, cardiac MRI, myocardium, cardiac, and cardiovascular. (Supplemental Methods). After screening all titles and abstracts in the search results, potentially relevant studies were reviewed in full by two reviewers (S.K. and N.Y.) for eligibility. Discrepancies were resolved by consensus. We registered our study protocol with the University Medical Information Network (registration number: 000055257). Institutional review board approval was not obtained for this study because it is a meta-analysis, and therefore, does not deal with clinical patient information. Studies including synthetic ECV data for various cardiac diseases and healthy control were included for data extraction. We also extracted laboratory ECV as reference data from the literature. Literatures such as case reports, animal studies, and non-English articles were excluded.

### 2.2 Outcome measures

The primary outcome of this meta-analysis was the estimation of synthetic ECV values and a comparison of laboratory ECV in different modalities. Two reviewers (S.K. and N.Y.) independently extracted the following characteristics from each study: author names, year of publication, country, sample size, patient demographics, MRI scanner type, protocol, and laboratory ECV and synthetic ECV values, correlation. We performed meta-analyses on the correlation coefficients between synthetic ECV and laboratory ECV. We used the Newcastle-Ottawa Quality Assessment Scale and case control studies to evaluate the risk of bias [12].

### 2.3 Data synthesis and statistical analyses

The meta-analysis was conducted using RevMan 5.41 (Cochrane Collaboration, London, UK) and R Statistical Software (v3.5.1, R Foundation for Statistical Computing, Vienna, Austria). A random-effects model was applied to estimate the pooled synthetic ECV values. For weighting each study in the meta-analysis, the inverse variance method was used. The evaluation of bias of these ECV measurements was performed by calculating the mean absolute difference. Heterogeneity was indicated by I^2^, where 0% meant no heterogeneity and 100% meant strong heterogeneity [13]. A summary receiver operating characteristic (sROC) curve was constructed to evaluate the accuracy of synthetic ECV compared to the laboratory normal cutoff of ECV. A funnel plot was created to assess publication bias, and statistical evaluation was performed using Begg's test. A p-value of <0.10 was considered statistically significant for Begg's test, while a p-value of <0.05 was defined as statistically significant for other statistical tests.

## 3. Results

**Fig. 1:**
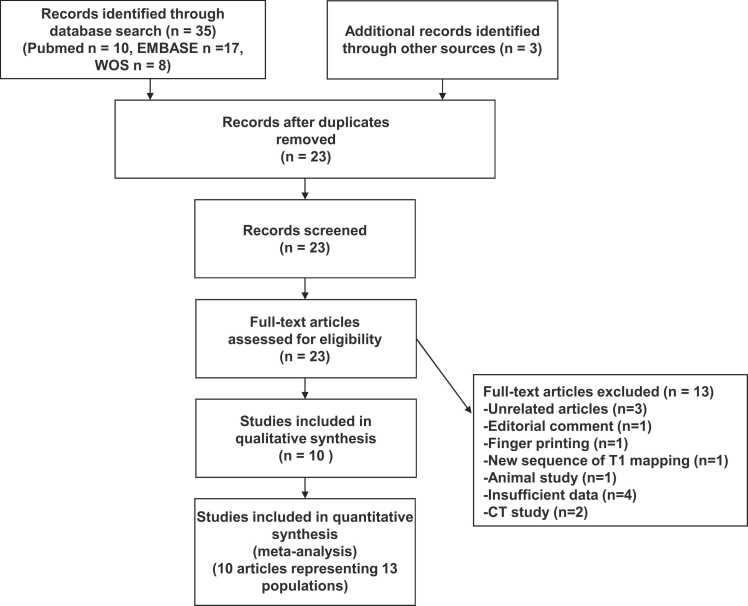
Preferred reporting items for systematic reviews and meta-analyses flow diagram. *WOS* web of science, *CT* computed tomography

Finally, 10 eligible publications [10], [14], [15], [16], [17], [18], [19], [20], [21], [22] were selected from 38 candidate papers (Fig. 1) and the characteristics of the included studies are summarized in Table 1, Table 2. Thirteen populations were presented in these 10 publications. Patients were classified into two categories for analysis: 1.5T MRI, [10], [16], [18], [19], [20], [22] 3.0T MRI, [14], [15], [16], [17], [21]. The publication years ranged from 2016 to 2024. This meta-analysis included 4492 cases for MRI (including 2525 cases of 1.5T MRI and 1967 cases of 3.0T MRI). All studies for MRI in the literature used modified Look-Locker inversion recovery (MOLLI) as the pulse sequence. The quality ratings of the studies for the assessment of risk of bias are summarized in Supplemental Table 1. Overall, 7 of 10 studies (70%) were rated as high quality (scoring more than 80% on the quality scales), 3 of 10 studies (30%) were rated as moderate quality (scoring between 50% and 80% on the quality scales), and none of 10 studies (0%) were rated as low quality (less than 50% score on the scales). A funnel plot for assessing publication bias in synthetic MRI-ECV and laboratory MRI-ECV is depicted in Supplemental Figure 1. No significant publication bias was detected by Begg's test (p = 0.76).

**Table 1:** characteristics of included populations.

First author	Year	ref #	Country	Inclusion criteria	N	Age (years)	Male. %	CMR/CT scanner
*MRI*								
Yin	2024	14	China	ICM, DCM, HCM, Myocarditis, Arrhythmia, CTD, hypertensive cardiomyopathy, CA, cardiac tumors, VHD, PH, normal heart	426	50.5 ± 15.7	60.3	3.0T MAGNETOM Skyra, Siemens
Reiter	2024	15	Austria	consecutive CMR scans routinely performed on a 3.0T scanner	80	N/A	N/A	3.0T MAGNETOM Skyra, Siemens
Chen	2022	16	Germany	written informed consent and completion of contrast CMR	326 (3.0T) 225 (1.5T)	52.1 ± 16.5 52.2 ± 17.1	62.0 59.1	3.0T Ingenia, Philips 1.5T Achieva, Philips
Thongsongsang	2021	17	Thailand	CA, DCM, HCM, myocarditis, CAD, healthy control	909	N/A	N/A	3.0T Ingenia, Philips
Censi	2021	18	Italy	healthy control, HCM, hypertensive subjects, athletes, CA, AS, DCM, HNDC	165	54 (43–67)	70	1.5T Ingenia, Philips
Robison	2018	19	Canada	chemotoxicity, HCM, AFD, myocarditis/pericarditis, CS, cardiomyopathy (cause unspecified), healthy subjects	74	N/A	N/A	1.5T MAGNETOM Avanto, Siemens
Kammerlander	2018	20	Austria	HF, VHD, CAD, others	513	57.4 ± 17.5	50	1.5T MAGNETOM Avanto, Siemens
Shang	2018	21	China	T2DM, HCM, healthy control, others	226	N/A	57.9	3.0T MAGNETOM Trio, Siemens
Raucci	2017	22	USA	congenital, secondary, acquired heart disease, normal heart	163	16.4 ± 6.4	89	1.5T Avanto, Siemens
Treibel	2016	10	UK	Validation cohort (healthy volunteer, AS, CA, HCM, patients post-anthracycline chemotherapy) Outcome cohort	213 1172	60 ± 15 56 (43–66)	48.8 52	1.5T MAGNETOM Avanto, Espree, and Aera, Siemens

Values for age are mean ± SD, median (IQR)

*CMR* cardiovascular magnetic resonance, *CT* computed tomography, *ICM* ischemic cardiomyopathy, *DCM* dilated cardiomyopathy, *HCM* hypertrophic cardiomyopathy, *CTD* connective tissue disease, *CA* cardiac amyloidosis, *VHD* valvular heart disease, *PH* pulmonary hypertension, *CAD* coronary artery disease, *T2DM* type 2 diabetes mellitus, *AS* aortic stenosis, *HNDC* hypokinetic non dilated cardiomyopathy, *AFD* Anderson-Fabry disease, *CS* cardiac sarcoidosis, *HF* heart failure; *N/A* not applicable

**Table 2:** Imaging characteristics.

First author, Year_subgroup	ref #	Lab-ECV	Syn-ECV	*N*	Correlation coefficient	blood pool, location	ECV, location	Contrast dose	Post-contrast timing (min)	Hct measurement timing
*MRI*										
Yin, 2024	14	28.3 (24.8–32.1)	28.2 (25.0–32.0)	426	0.93	LV	septum	0.2 mmol/kg (Gd-DTPA)	15–20	within 24 h
Reiter, 2024	15	28.7 ± 5.9	N/A	80	0.94	LV	septum	N/A	10	within 24 h
Chen, 2022	16									
Chen,2022_validation (1.5T)		N/A	N/A	225	0.93	LV	septum	0.15 mmol/kg (Gadobutrol)	15	within 24 h
Chen,2022_validation (3.0T)		N/A	N/A	326	0.94	LV	septum	0.15 mmol/kg (Gadobutrol)	15	within 24 h
Thongsongsang, 2021	17	N/A	N/A	909	0.84	LV	septum	0.15 mmol/kg (gadoterate meglumine, Gadobutrol, gadopentetate dimeglumine)	10	on the day of or within 6 months
Thongsongsang, 2021_amyloidosis		47.2 ± 10.5	49.3 ± 11.8	8	N/A	LV	septum			
Thongsongsang, 2021_DCM		29.9 ± 4.5	30.0±3.9	116	N/A	LV	septum			
Thongsongsang, 2021_HCM		30.1 ± 4.6	31.1±5.8	84	N/A	LV	septum			
Thongsongsang, 2021_myocarditis		30.5 ± 5.7	31.3±5.3	13	N/A	LV	septum			
Thongsongsang, 2021_CAD		29.3 ± 5.5	29.1±4.9	223	N/A	LV	septum			
Thongsongsang, 2021_control		27.9 ± 3.6	27.0±3.3	465	N/A	LV	septum			
Censi, 2021	18	N/A	N/A	165	0.97	LV	septum	0.15 mmol/kg (gadoterate meglumine, gadopentate dimeglumine)	10–30	immediately after the scan
Censi, 2021_healthy control		27 ± 2	26±2	52	N/A	LV	septum			
Censi, 2021_HCM		30 ± 4	29 (27–30)	21	N/A	LV	septum			
Censi, 2021_hypertensive subjects		27 ± 3	26 (23–27)	18	N/A	LV	septum			
Censi, 2021_athletes		26 ± 1	25 ± 2	15	N/A	LV	septum			
Censi, 2021_CA		46 ± 10	44 ± 9	11	N/A	LV	septum			
Censi, 2021_AS		30 ± 4	29 ± 5	9	N/A	LV	septum			
Censi, 2021_DCM		30 ± 3	28 ± 3	17	N/A	LV	septum			
Censi, 2021_HNDC		28 ± 3	27 ± 3	20	N/A	LV	septum			
Robison, 2018	19	27.1 ± 4.7	26.5 ± 4.3	74	0.92	LV	LV myocardium	0.15 mmol/kg (gadobuterol)	12–15	within 24 h (median 1.3 h)
Kammerlander, 2018	20	28.4 ± 6.8	28.3 ± 6.6	513	0.959	LV	mid LV myocardium	0.1 mmol/kg (gadobutrol)	N/A	prior to the contrast agent administration
Shang, 2018	21							0.2 mmol/kg (gadoteric acid meglumine bolus)	15	N/A
Shang, 2018_derivation		N/A	N/A	121	0.93	LV	mid LV myocardium			
Shang, 2018_derivation (healthy control)		26.4 ± 2.4	26.9 ± 2.2	34	N/A	LV	mid LV myocardium			
Shang, 2018_derivation (T2DM)		29.1 ± 3.1	28.3 ± 2.9	18	N/A	LV	mid LV myocardium			
Shang, 2018_derivation (HCM)		30.0 ± 5.0	30.1 ± 4.2	49	N/A	LV	mid LV myocardium			
Shang, 2018_validation		N/A	N/A	105	0.84	LV	mid LV myocardium			
Shang, 2018_validation (healthy control)		25.8 ± 3.2	26.7 ± 2.6	26	N/A	LV	mid LV myocardium			
Shang, 2018_validation (T2DM)		28.6 ± 2.9	28.0 ± 2.3	27	N/A	LV	mid LV myocardium			
Shang, 2018_validation (HCM)		30.1 ± 4.3	29.8 ± 4.4	44	N/A	LV	mid LV myocardium			
Raucci, 2017	22	29.5 ± 3.9	29.5 ± 3.6	163	0.91	N/A	septum	0.2 mmol/kg (gadopentate dimeglumine, gadobutrol)	15	within 3 months
Treibel, 2016	10									
Treibel, 2016_validation		33 ± 11	N/A	213	0.98	LV	septum	0.1 mmol/kg (gadoterate meglumine)	15–20	N/A
Treibel, 2016_outcome		28 (26–31)	N/A	1172	0.91	LV	septum	0.2 mmol/kg (gadoteridol)	15–20	N/A

Values for ECV are mean ± SD, median (IQR).

*ECV* extracellular volume fraction, *Lab* laboratory, *Syn* synthetic, *Hct* hematocrit, *Gd-GTPA* gadolinium-diethylenetriaminepentaacetic acid, *LV* left ventricular, *CMR* cardiac magnetic resonance, *CT* computed tomography, *ICM* ischemic cardiomyopathy, *DCM* dilated cardiomyopathy, *HCM* hypertrophic cardiomyopathy, *CTD* connective tissue disease, *CA* cardiac amyloidosis, *VHD* valvular heart disease, *PH* pulmonary hypertension, *CAD* coronary artery disease, *T2DM* type 2 diabetes mellitus, *AS* aortic stenosis, *HNDC* hypokinetic non dilated cardiomyopathy, *AFD* Anderson-Fabry disease, *CS* cardiac sarcoidosis, *HF* heart failure; *N/A* not applicable

### 3.1 Synthetic ECV vs laboratory ECV for CMR

**Fig. 2:**
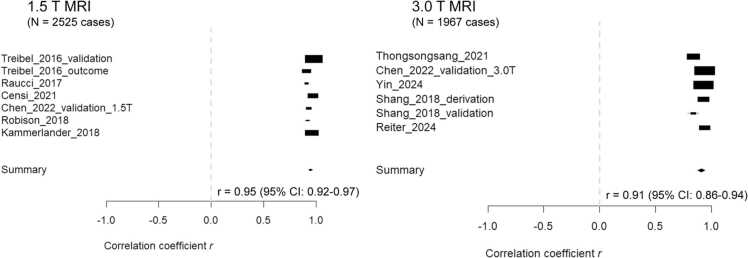
Forest plot of correlation coefficient between synthetic ECV and laboratory ECV for CMR Forest plot of correlation coefficients between synthetic ECV and laboratory ECV. A high correlation between synthetic ECV and laboratory ECV is observed, reflected by a summary correlation coefficient of 0.95 (95%CI: 0.92 to 0.97, p<0.001) at 1.5T MRI and 0.91 (95%CI: 0.86 to 0.94, p<0.001) at 3T MRI. *CMR* cardiac magnetic resonance, *ECV* extracellular volume fraction.

Fig. 2 shows the results of the meta-analysis of correlation coefficients between synthetic ECV and laboratory ECV in different strength CMR: ten studies provided correlation coefficients [10], [14], [15], [16], [17], [18], [19], [20], [21], [22]. An almost perfect correlation was observed between synthetic ECV and laboratory ECV [summary correlation coefficient: 0.95 (95%CI: 0.92 to 0.97, p<0.001) at 1.5T MRI and 0.91 (95%CI: 0.86 to 0.94, p<0.001) at 3.0T MRI].

**Fig. 3:**
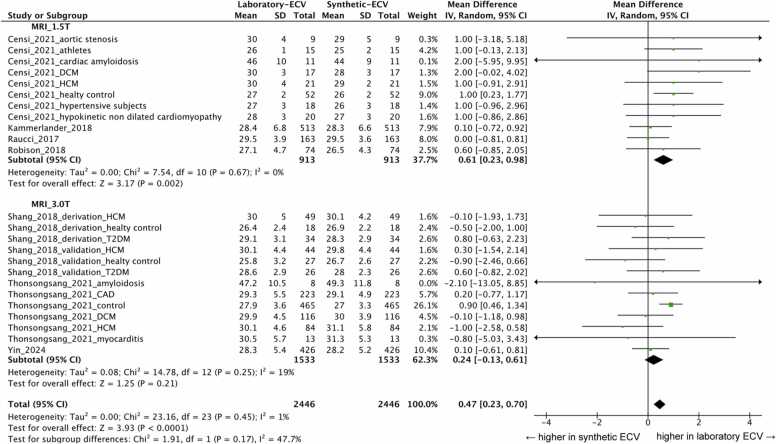
Forest plot of mean difference between synthetic ECV and laboratory ECV for CMR The meta-analysis shows that mean difference between synthetic ECV and laboratory ECV was minimal (mean difference = 0.61; 95% CI, 0.23 to 0.98; I^2^ = 0%; p for heterogeneity = 0.67) at 1.5T MRI and (mean difference = 0.24; 95% CI, −0.13 to 0.61; I^2^ = 19%; p for heterogeneity = 0.25) at 3.0 T MRI. *IV* inverse variance, *CMR* cardiac magnetic resonance, *ECV* extracellular volume fraction, *MRI* magnetic resonance imaging, *CI* confidence interval

Fig. 3 presents the meta-analysis results for synthetic ECV across different CMR field strengths. The bias between laboratory ECV and synthetic ECV was minimal, with a mean difference of 0.61% (95% CI: 0.23 to 0.98%, I² = 0%, p for heterogeneity = 0.67) at 1.5T MRI and 0.24% (95% CI: −0.13 to 0.61%, I² = 19%, p for heterogeneity = 0.25) at 3.0T MRI. In a subgroup analysis, the included studies are divided into three subgroups: healthy control (normal ECV), patients with cardiovascular disease (mild abnormalities), and those with cardiac amyloidosis (highly elevated ECV). No significant differences were observed among the groups at both 1.5T and 3.0T MRI (p = 0.68 and 0.92, respectively), as shown in Supplemental Figs. 2 and 3. Additionally, a separate subgroup analysis was performed for the healthy cohort and those with cardiovascular disease. A statistical comparison between these two groups revealed no significant differences at both 1.5T and 3.0T MRI (p = 0.41 and 0.89, respectively), as shown in Supplemental Figs. 4 and 5.

We performed a meta-analysis focusing on the diagnostic accuracy of synthetic ECV using laboratory ECV cutoff values, based on two studies (which include three subgroups) [16], [21]. The summary Receiver Operating Characteristic (sROC) curve showed an excellent diagnostic performance, with an AUC of 0.98. Additionally, the sensitivity was 0.85 (95% CI: 0.79 to 0.90), and the specificity was 0.97 (95% CI: 0.94 to 0.98) (see Supplementary Fig. 6).

## 4. Discussion

Our study, which analyzed data from 4492 patients across 13 populations, is the first comprehensive systematic review and meta-analysis to evaluate the correlation and agreement between synthetic ECV and laboratory ECV in CMR. In the present study, we found an excellent correlation between synthetic ECV and laboratory ECV. The agreement between the two was also high, with a mean difference in ECV values of less than 1%. Despite the diversity of studies in terms of multiple MRI vendors, patient demographics, such as age and various underlying pathologies (e.g., myocardial infarction, cardiomyopathy), the correlation between synthetic and laboratory ECV remained consistently strong. This suggests that synthetic ECV can be effectively applied across different populations and clinical conditions. This high level of agreement indicates that the lack of blood sampling for Hct in clinical routine or retrospective studies is no longer a significant barrier to calculating ECV. Additionally, synthetic ECV map can be generated online during image acquisition, offering convenience in obtaining the ECV in the future.

MRI-derived ECV is a well-established noninvasive biomarker that correlates with pathologies including extracellular edema, diffuse interstitial fibrosis, abnormal infiltration of the myocardium by amyloid protein, or lipids, and predicts mortality and major adverse events independently as well as left ventricular ejection fraction [3], [23], [24], [25]. Current methods for calculating ECV rely on blood Hct measurements, which are cumbersome and time-consuming, thereby posing a significant barrier to the routine implementation of this technique in clinical practice. The high correlation between synthetic ECV and laboratory ECV at CMR has been demonstrated in both our study and existing research. Additionally, synthetic ECV of CMR and the histologic gold standard collagen volume fraction were well correlated (R^2^ = 0.61) and synthetic ECV was significantly associated with increased risk of hospitalization for heart failure or death [10]. Thus, avoiding venous blood sampling for ECV calculation would further facilitate the widespread use of this imaging biomarker.

Under physiological conditions, blood is composed mainly of plasma and erythrocytes, with the erythrocyte volume fraction referred to as Hct. To date, the longitudinal relaxation rate of water in blood has been measured in vivo and in vitro at multiple magnetic fields, showing a linear dependence of longitudinal relaxation rate (R1 = 1/T1) on Hct. Based on this linear relationship between the R1 of the blood pool and Hct, a site-specific formula for R1 and synthetic Hct is derived [26], [27], [28]. Other factors including oxygen saturation, body temperature are also known to affect blood T1 [29]. While blood Hct within the normal range is a strong determinant of blood pool T1 time, lower and higher laboratory Hct were both related to the miscalculation of ECV. This may be because in addition to Hct, other factors such as iron, high-density lipoprotein cholesterol determine the T1 values of the blood pool [30]. Therefore, especially in critically ill patients or patients with conditions causing a grossly abnormal blood composition, synthetic Hct from blood pool T1 time may provide an unreliable estimate, and clinical decisions based on this synthetic method should be made with caution.

Although the correlation of synthetic Hct and laboratory Hct is only moderate, synthetic ECV performance is almost perfect. This may be due to the inclusion of four additional factors, including native and post-contrast T1 of the myocardium and blood, in the ECV calculation. Regarding the calculation of synthetic ECV and laboratory ECV, except for blood Hct, these four factors remained unchanged, which may reduce the impact of Hct variability on the ECV calculation. Another reason for the higher correlation between synthetic and laboratory ECV compared to the correlation between laboratory and synthetic Hct is that R1 is a direct measurement of the blood pool in the left ventricular cavity, which may be considered more reliable than Hct measurements obtained from peripheral blood sampling [18], [21], [22], [31]. However, synthetic ECV remains controversial due to the possibility of misclassification rates of synthetic ECV for patients with borderline ECV. In our meta-analysis, although the number of studies was limited, it was demonstrated that the synthetic ECV classified normal and abnormal cases based on laboratory ECV cutoff with high accuracy. In contrast, some studies reported misclassification rates of synthetic ECV of 12 to 25% and concluded this may lead to relevant clinical errors [21], [22]. We believe this high misclassification rate is in part due to variability in laboratory Hct when taken as a routine clinical test and may be reduced by performing blood sampling in a standardized way such as the timing of Hct measurement [32], [33].

As with other non-contrast mapping parameters, synthetic Hct necessitates local regression model, unless MRI scanners, imaging acquisition parameters, contrast agent injection protocol, and Hct measurement device are identical. Previous systematic review that showed native T1 measurements in healthy adults greatly depends on a variety of factors such as differences in center-specific protocols, population studied, vendor, and pulse sequence [34]. Different formulas for derivation of synthetic Hct have been described in the literature. Reiter et al. showed synthetic ECV at 3.0T scanner calculated by published formula that were derived from a different 3.0T scanner resulted in leading a misclassification up to 60%. These results suggest that using published formulas for calculating synthetic ECV may lead to significant errors, even when derived from sites employing similar hardware and scanning protocols. Therefore, to minimize intra- and interpatient variability, it is essential for each institution to develop site-specific formulas for calculating synthetic ECV, similar to site-specific normal values for T1 times, before incorporating synthetic ECV into routine clinical practice.

## 5. Limitations

This study has some limitations. First, many of the studies included in this meta-analysis were retrospective, single-center observational studies, and there was a lack of large prospective studies. Each study's selection bias cannot be ignored. Second, we chose to only include studies that used MOLLI as the pulse sequence scheme because of the dearth of other schemes in the literature. However, most of the studies reported in the literature used a MOLLI-based pulse sequence, and thus this meta-analysis provided an assessment of the ECV by using these clinically available pulse sequences. Third, patient factors include the presence of comorbidities that affect ECV, such as hypertension and diabetes. Methodology also varies, and including the type of scanner, location of region of interest drawn in the myocardium and blood pool, contrast dose, timing of blood Hct sampling and imaging after administration of contrast agent. It can be assumed the significant heterogeneity was observed in the present study due to these causes, and these issues need to be recognized if synthetic ECV is to be properly interpreted for clinical use. Fourth, this meta-analysis integrated synthetic ECV results derived from different predictive models, and the variability between these models may impact the comparability and pooling of results. Each model has distinct characteristics, assumptions, and datasets, which may lead to discrepancies in results. Despite these differences in local models, the heterogeneity across studies was low, and a random-effects model was used to account for potential variability between models and to address any inherent biases. Future studies should focus on standardizing predictive models and validating them across different clinical populations and MRI protocols to improve comparability and clinical applicability. Fifth, in the subgroup analysis of this meta-analysis, the population of cardiac amyloidosis patients is small at both 1.5T and 3T, and a comprehensive evaluation could not be conducted. Accurate ECV quantification and changes in ECV are emerging as important prognostic markers in cardiac amyloidosis [35]. Therefore, further research is needed to assess the diagnostic performance of synthetic ECV in cardiac amyloidosis. Finally, while this study concentrated on evaluating the correlation and agreement between synthetic ECV and laboratory ECV, it did not address the optimal threshold of synthetic ECV for detecting significant myocardial fibrosis or predicting cardiovascular outcomes.

## 6. Conclusions

Our study is the first comprehensive systematic review and meta-analysis of synthetic ECV. Synthetic ECV showed excellent correlation with laboratory ECV at CMR. The mean difference between synthetic ECV and laboratory ECV was <1% at CMR. Despite the necessity for further validation, the synthetic ECV calculated using estimates of the Hct derived from blood T1 may be a convenient and concise tool to evaluate diffuse myocardial fibrosis or other infiltrative myocardial diseases in clinical practice.

## Funding

Japan Society for the Promotion of Science, Grant-in-Aid for Scientific Research (C).

## Author contributions

**Shungo Sawamura**: Data curation, Conceptualization. **Hiroaki Nagase**: Formal analysis, Data curation, Conceptualization. **Daisuke Utsunomiya**: Supervision. **Naofumi Yasuda**: Software, Resources, Project administration, Methodology, Investigation, Funding acquisition, Formal analysis, Data curation, Conceptualization. **Shingo Kato**: Funding acquisition, Formal analysis, Data curation, Conceptualization. **Nobuyuki Horita**: Writing – review & editing, Writing – original draft, Visualization, Validation, Supervision. **Ryusuke Sekii**: Funding acquisition, Formal analysis, Data curation, Conceptualization.

## Declaration of competing interests

The authors declare that they have no known competing financial interests or personal relationships that could have appeared to influence the work reported in this paper.
