# Supplementary material for: Synthetic extracellular volume fraction as an imaging biomarker of the myocardial interstitium without blood sampling: a systematic review and meta-analysis
Source: J Cardiovasc Magn Reson. 2025 Mar 24;27(1):101889. doi: 10.1016/j.jocmr.2025.101889 (PMC12138551; doi:10.1016/j.jocmr.2025.101889)
Supplement: Supplementary file 1 — Supplementary material [file mmc1.pdf]

Supplemental Methods

PubMed 10

#1 (synthetic extracellular volume fraction[Title/Abstract] OR synthetic ECV[Title/Abstract])

#2 (cardiac computed tomography[Title/Abstract] OR cardiac CT[Title/Abstract] OR cardiac MRI[Title/Abstract] OR cardiac magnetic resonance[Title/Abstract])

#3 (Myocardium[Title/Abstract] OR Cardiac[Title/Abstract] OR Cardiovascular[Title/Abstract])

#4 (#1 AND #2 AND #3)

WOS 17

#1 TS=(synthetic extracellular volume fraction OR synthetic ECV)

#2 TS=(cardiac computed tomography OR cardiac CT OR cardiac MRI OR cardiac magnetic resonance)

#3 TS=(Myocardium OR Cardiac OR Cardiovascular)

#4 #1 AND #2 AND #3

EMBASE 8

TITLE: (synthetic extracellular volume fraction OR synthetic ECV) AND

(cardiac computed tomography OR cardiac CT OR cardiac MRI OR cardiac magnetic resonance) AND

(Myocardium OR Cardiac OR Cardiovascular)

Supplemental Table 1

NEWCASTLE - OTTAWA QUALITY ASSESSMENT SCALE CASE CONTROL STUDIES

**Study**	**Selection (Max=4)**	**Comparability (Max=2)**	**Exposure (Max=3)**	**Total Score**
Treibel2016	3	1	3	7
Raucci2017	3	2	3	8
Robison2018	3	2	3	8
Kammerlander2018	3	2	3	8
Shang2018	3	2	3	8
Censi2021	3	2	3	8
Thongsongsang2021	3	2	3	8
Chen2022	3	1	3	7
Yin2024	3	2	3	8
Reiter2024	3	1	3	7

Supplemental Figure 1

Funnel plot of the mean difference of synthetic MRI-ECV and laboratory ECV

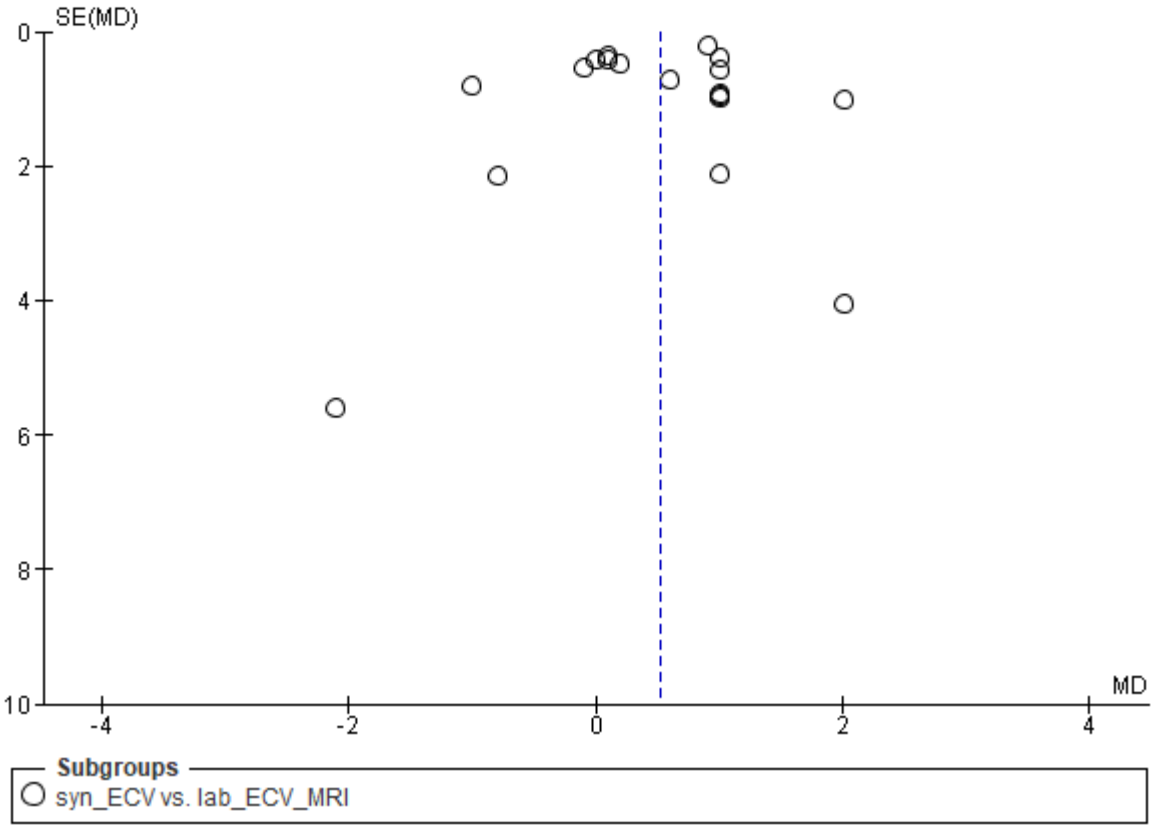

Rank Correlation Test for Funnel Plot Asymmetry

Kendall's tau = -0.0588, p = 0.7652

Supplemental Figure 2

Forrest Plot of Mean Difference Between Laboratory ECV and Synthetic ECV on 1.5 T MRI (Subgroup Analysis: healthy vs amyloidosis vs cardiovascular disease)

	Laboratory-ECV			Synthetic-ECV				Mean Difference	Mean Difference
**Study or Subgroup**	**Mean**	**SD**	**Total**	**Mean**	**SD**	**Total**	**Weight**	**IV, Random, 95% CI**	**IV, Random, 95% CI**
**3.5.1 MRI_1.5T_healthy**									
Censi_2021_healty control	27	2	52	26	2	52	39.2%	1.00 [0.23, 1.77]	
**Subtotal (95% CI)**			**52**			**52**	**39.2%**	**1.00 [0.23, 1.77]**	
Heterogeneity: Not applicable									
Test for overall effect: Z = 2.55 (P = 0.01)									
**3.5.2 MRI_1.5T_amyloidosis**									
Censi_2021_cardiac amyloidosis	46	10	11	44	9	11	0.4%	2.00 [−5.95, 9.95]	
**Subtotal (95% CI)**			**11**			**11**	**0.4%**	**2.00 [−5.95, 9.95]**	
Heterogeneity: Not applicable									
Test for overall effect: Z = 0.49 (P = 0.62)									
**3.5.3 MRI_1.5T_CVD**									
Censi_2021_aortic stenosis	30	4	9	29	5	9	1.3%	1.00 [−3.18, 5.18]	
Censi_2021_DCM	30	3	17	28	3	17	5.7%	2.00 [−0.02, 4.02]	
Censi_2021_HCM	30	4	21	29	2	21	6.3%	1.00 [−0.91, 2.91]	
Censi_2021_hypertensive subjects	27	3	18	26	3	18	6.0%	1.00 [−0.96, 2.96]	
Censi_2021_hypokinetic non dilated cardiomyopathy	28	3	20	27	3	20	6.7%	1.00 [−0.86, 2.86]	
Kammerlander_2018	28.4	6.8	513	28.3	6.6	513	34.4%	0.10 [−0.72, 0.92]	
**Subtotal (95% CI)**			**598**			**598**	**60.5%**	**0.58 [−0.04, 1.20]**	
Heterogeneity: $Tau^2 = 0.00$; $Chi^2 = 3.82$, df = 5 (P = 0.58); $I^2 = 0\%$									
Test for overall effect: Z = 1.84 (P = 0.07)									
**Total (95% CI)**			**661**			**661**	**100.0%**	**0.75 [0.27, 1.23]**	
Heterogeneity: $Tau^2 = 0.00$; $Chi^2 = 4.60$, df = 7 (P = 0.71); $I^2 = 0\%$									
Test for overall effect: Z = 3.06 (P = 0.002)									
Test for subgroup differences: $Chi^2 = 0.78$, df = 2 (P = 0.68), $I^2 = 0\%$									

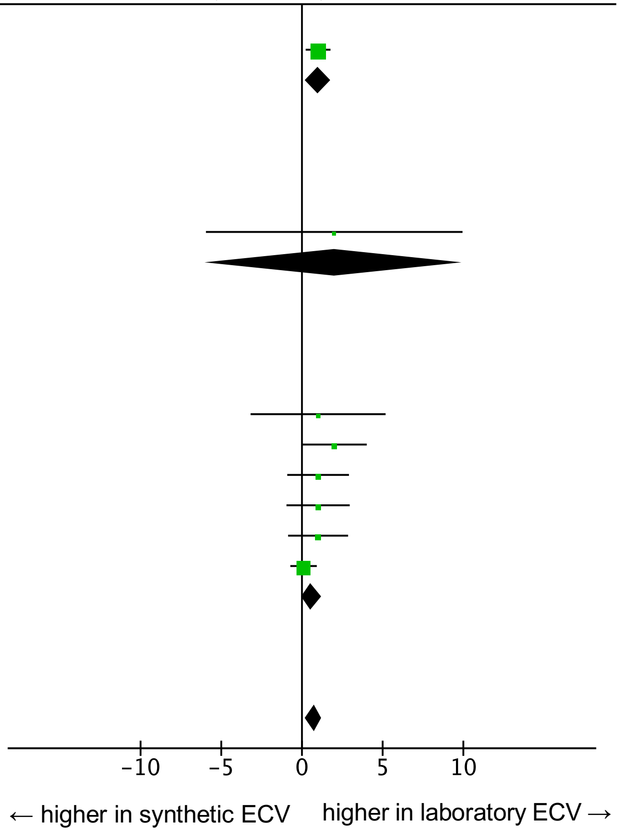

Supplemental Figure 3

Forrest Plot of Mean Difference Between Laboratory ECV and Synthetic ECV on 3.0 T MRI (Subgroup Analysis: healthy vs amyloidosis vs cardiovascular disease)

Study or Subgroup	Laboratory-ECV Mean	Laboratory-ECV SD	Laboratory-ECV Total	Synthetic-ECV Mean	Synthetic-ECV SD	Synthetic-ECV Total	Weight	Mean Difference IV, Random, 95% CI
**3.6.1 MRI_3.0T_healthy**								
Shang_2018_derivation_healty control	26.4	2.4	18	26.9	2.2	18	6.9%	−0.50 [−2.00, 1.00]
Shang_2018_validation_healty control	25.8	3.2	27	26.7	2.6	27	6.5%	−0.90 [−2.46, 0.66]
Thonsongsang_2021_control	27.9	3.6	465	27	3.3	465	29.3%	0.90 [0.46, 1.34]
**Subtotal (95% CI)**			**510**			**510**	**42.7%**	**0.01 [−1.26, 1.28]**
Heterogeneity: $Tau^2 = 0.90$; $Chi^2 = 7.27$, df = 2 (P = 0.03); $I^2 = 72\%$								
Test for overall effect: Z = 0.01 (P = 0.99)								
**3.6.2 MRI_3.0T_amyloidosis**								
Thonsongsang_2021_amyloidosis	47.2	10.5	8	49.3	11.8	8	0.2%	−2.10 [−13.05, 8.85]
**Subtotal (95% CI)**			**8**			**8**	**0.2%**	**−2.10 [−13.05, 8.85]**
Heterogeneity: Not applicable								
Test for overall effect: Z = 0.38 (P = 0.71)								
**3.6.3 MRI_3.0T_CVD**								
Shang_2018_derivation_HCM	30	5	49	30.1	4.2	49	4.9%	−0.10 [−1.93, 1.73]
Shang_2018_derivation_T2DM	29.1	3.1	34	28.3	2.9	34	7.5%	0.80 [−0.63, 2.23]
Shang_2018_validation_HCM	30.1	4.4	44	29.8	4.4	44	4.9%	0.30 [−1.54, 2.14]
Shang_2018_validation_T2DM	28.6	2.9	26	28	2.3	26	7.5%	0.60 [−0.82, 2.02]
Thonsongsang_2021_CAD	29.3	5.5	223	29.1	4.9	223	13.5%	0.20 [−0.77, 1.17]
Thonsongsang_2021_DCM	29.9	4.5	116	30	3.9	116	11.5%	−0.10 [−1.18, 0.98]
Thonsongsang_2021_HCM	30.1	4.6	84	31.1	5.8	84	6.3%	−1.00 [−2.58, 0.58]
Thonsongsang_2021_myocarditis	30.5	5.7	13	31.3	5.3	13	1.0%	−0.80 [−5.03, 3.43]
**Subtotal (95% CI)**			**589**			**589**	**57.1%**	**0.11 [−0.39, 0.61]**
Heterogeneity: $Tau^2 = 0.00$; $Chi^2 = 3.69$, df = 7 (P = 0.81); $I^2 = 0\%$								
Test for overall effect: Z = 0.43 (P = 0.67)								
**Total (95% CI)**			**1107**			**1107**	**100.0%**	**0.23 [−0.20, 0.66]**
Heterogeneity: $Tau^2 = 0.11$; $Chi^2 = 14.03$, df = 11 (P = 0.23); $I^2 = 22\%$								
Test for overall effect: Z = 1.04 (P = 0.30)								
Test for subgroup differences: $Chi^2 = 0.17$, df = 2 (P = 0.92), $I^2 = 0\%$								

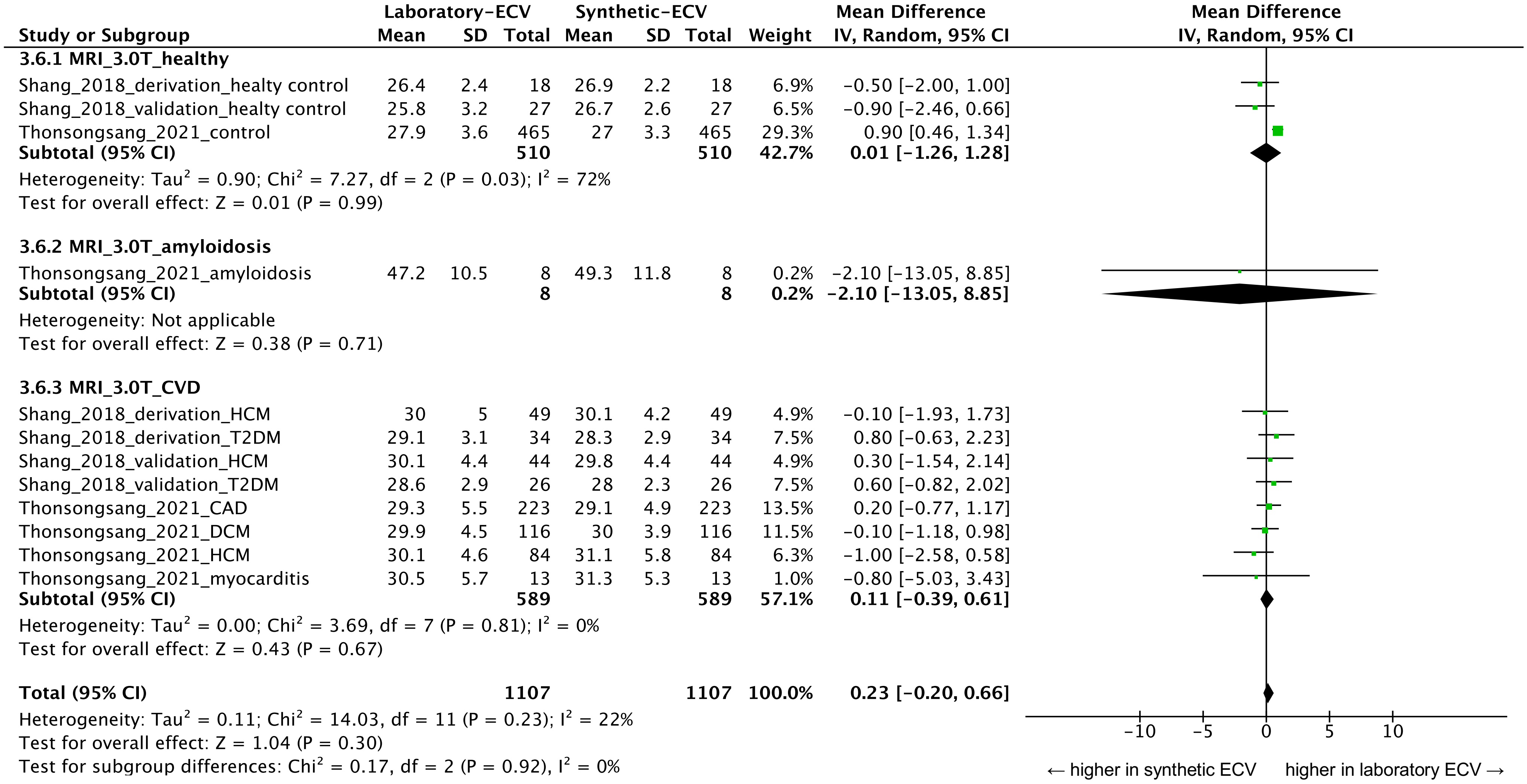

Supplemental Figure 4

Forrest Plot of Mean Difference Between Laboratory ECV and Synthetic ECV on 1.5 T MRI (Subgroup Analysis: healthy vs cardiovascular disease)

	Laboratory-ECV			Synthetic-ECV				Mean Difference
Study or Subgroup	Mean	SD	Total	Mean	SD	Total	Weight	IV, Random, 95% CI
**3.7.1 MRI_1.5T_healthy**								
Censi_2021_healty control	27	2	52	26	2	52	39.3%	1.00 [0.23, 1.77]
**Subtotal (95% CI)**			**52**			**52**	**39.3%**	**1.00 [0.23, 1.77]**
Heterogeneity: Not applicable								
Test for overall effect: Z = 2.55 (P = 0.01)								
**3.7.3 MRI_1.5T_CVD**								
Censi_2021_aortic stenosis	30	4	9	29	5	9	1.3%	1.00 [−3.18, 5.18]
Censi_2021_DCM	30	3	17	28	3	17	5.7%	2.00 [−0.02, 4.02]
Censi_2021_HCM	30	4	21	29	2	21	6.3%	1.00 [−0.91, 2.91]
Censi_2021_hypertensive subjects	27	3	18	26	3	18	6.0%	1.00 [−0.96, 2.96]
Censi_2021_hypokinetic non dilated cardiomyopathy	28	3	20	27	3	20	6.7%	1.00 [−0.86, 2.86]
Kammerlander_2018	28.4	6.8	513	28.3	6.6	513	34.5%	0.10 [−0.72, 0.92]
**Subtotal (95% CI)**			**598**			**598**	**60.7%**	**0.58 [−0.04, 1.20]**
Heterogeneity: $Tau^2 = 0.00$; $Chi^2 = 3.82$, df = 5 (P = 0.58); $I^2 = 0\%$								
Test for overall effect: Z = 1.84 (P = 0.07)								
**Total (95% CI)**			**650**			**650**	**100.0%**	**0.75 [0.26, 1.23]**
Heterogeneity: $Tau^2 = 0.00$; $Chi^2 = 4.51$, df = 6 (P = 0.61); $I^2 = 0\%$								
Test for overall effect: Z = 3.03 (P = 0.002)								
Test for subgroup differences: $Chi^2 = 0.69$, df = 1 (P = 0.41), $I^2 = 0\%$								

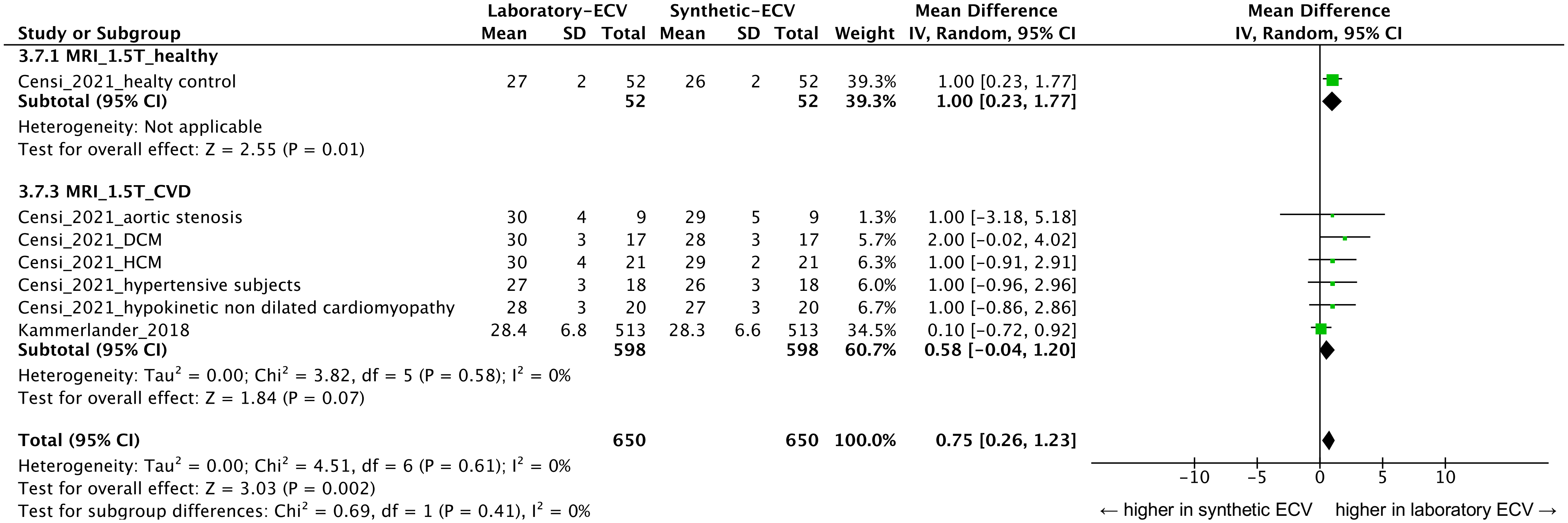

Supplemental Figure 5

Forrest Plot of Mean Difference Between Laboratory ECV and Synthetic ECV on 3.0 T MRI (Subgroup Analysis: healthy vs cardiovascular disease)

	Laboratory-ECV			Synthetic-ECV				Mean Difference
**Study or Subgroup**	**Mean**	**SD**	**Total**	**Mean**	**SD**	**Total**	**Weight**	**IV, Random, 95% CI**
**3.8.1 MRI_3.0T_healthy**								
Shang_2018_derivation_healty control	26.4	2.4	18	26.9	2.2	18	7.2%	-0.50 [-2.00, 1.00]
Shang_2018_validation_healty control	25.8	3.2	27	26.7	2.6	27	6.8%	-0.90 [-2.46, 0.66]
Thonsongsang_2021_control	27.9	3.6	465	27	3.3	465	27.1%	0.90 [0.46, 1.34]
**Subtotal (95% CI)**			**510**			**510**	**41.0%**	**0.01 [-1.26, 1.28]**
Heterogeneity: $Tau^2 = 0.90$; $Chi^2 = 7.27$, df = 2 (P = 0.03); $I^2 = 72\%$								
Test for overall effect: Z = 0.01 (P = 0.99)								
**3.8.3 MRI_3.0T_CVD**								
Shang_2018_derivation_HCM	30	5	49	30.1	4.2	49	5.2%	-0.10 [-1.93, 1.73]
Shang_2018_derivation_T2DM	29.1	3.1	34	28.3	2.9	34	7.8%	0.80 [-0.63, 2.23]
Shang_2018_validation_HCM	30.1	4.4	44	29.8	4.4	44	5.1%	0.30 [-1.54, 2.14]
Shang_2018_validation_T2DM	28.6	2.9	26	28	2.3	26	7.8%	0.60 [-0.82, 2.02]
Thonsongsang_2021_CAD	29.3	5.5	223	29.1	4.9	223	13.6%	0.20 [-0.77, 1.17]
Thonsongsang_2021_DCM	29.9	4.5	116	30	3.9	116	11.7%	-0.10 [-1.18, 0.98]
Thonsongsang_2021_HCM	30.1	4.6	84	31.1	5.8	84	6.6%	-1.00 [-2.58, 0.58]
Thonsongsang_2021_myocarditis	30.5	5.7	13	31.3	5.3	13	1.1%	-0.80 [-5.03, 3.43]
**Subtotal (95% CI)**			**589**			**589**	**59.0%**	**0.11 [-0.39, 0.61]**
Heterogeneity: $Tau^2 = 0.00$; $Chi^2 = 3.69$, df = 7 (P = 0.81); $I^2 = 0\%$								
Test for overall effect: Z = 0.43 (P = 0.67)								
**Total (95% CI)**			**1099**			**1099**	**100.0%**	**0.21 [-0.24, 0.66]**
Heterogeneity: $Tau^2 = 0.14$; $Chi^2 = 13.83$, df = 10 (P = 0.18); $I^2 = 28\%$								
Test for overall effect: Z = 0.90 (P = 0.37)								
Test for subgroup differences: $Chi^2 = 0.02$, df = 1 (P = 0.89), $I^2 = 0\%$								

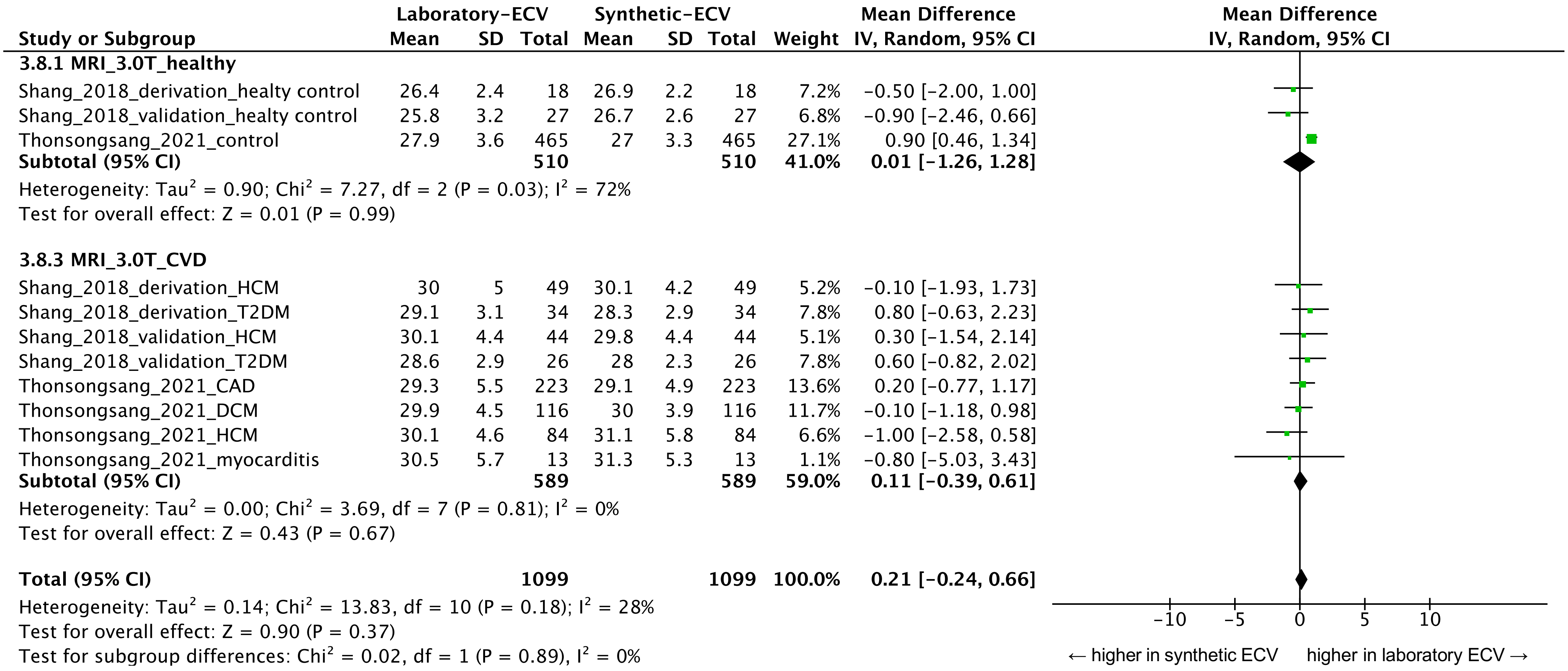

Supplemental Figure 6

Summary Receiver Operating Characteristic curve and pooled sensitivity and specificity of synthetic ECV in detecting abnormalities based on laboratory ECV cutoff.

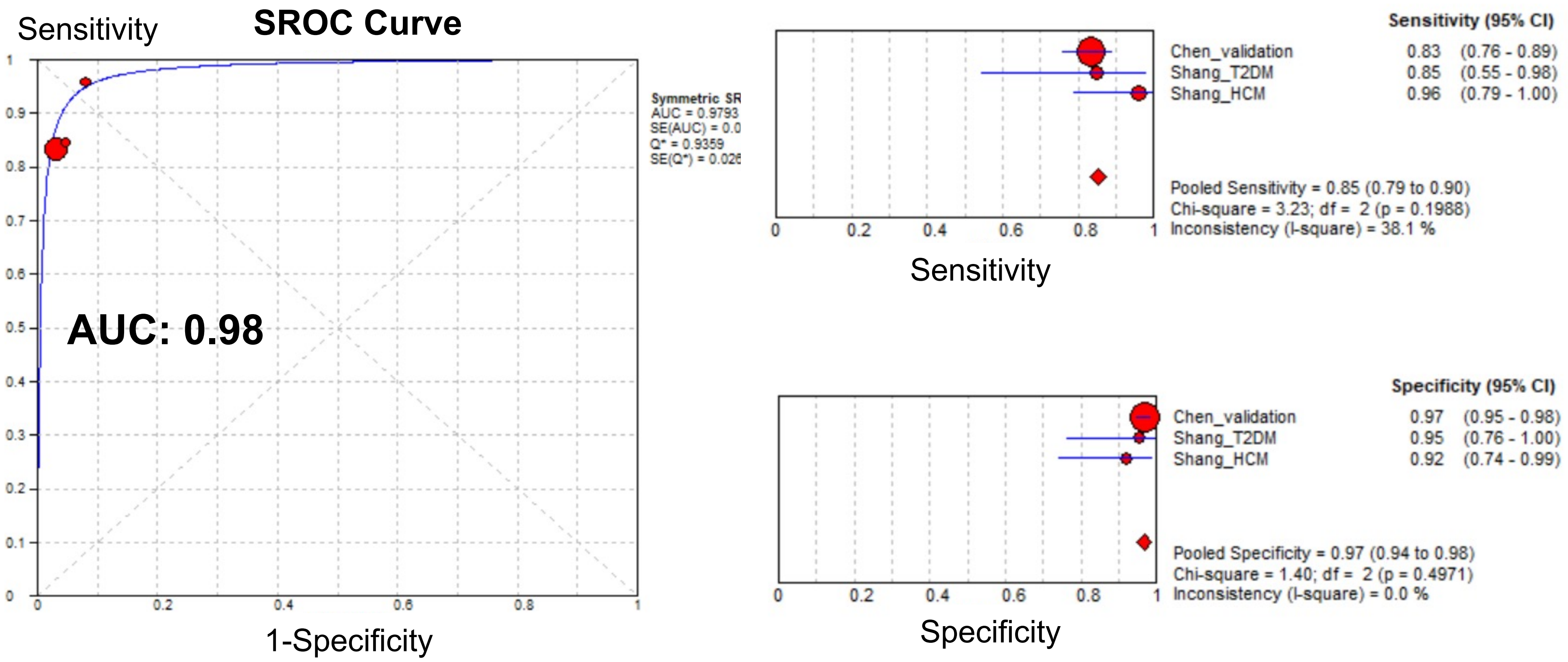

SROC = summary receiver operating characteristic, AUC = area under the curve
